# Relationships among Ocular Blood Flow Shown by Laser Speckle Flowgraphy, Retinal Arteriosclerotic Change, and Chorioretinal Circulation Time Obtained by Fluorescein Angiography

**DOI:** 10.1155/2017/2969064

**Published:** 2017-02-26

**Authors:** Hironori Osamura, Tomoaki Shiba, Takashi Itokawa, Tadashi Matsumoto, Yuichi Hori

**Affiliations:** Department of Ophthalmology, School of Medicine, Toho University, Tokyo, Japan

## Abstract

*Purpose*. To determine the correlations among the mean blur rate (MBR) in the optic nerve head (ONH) shown by laser speckle flowgraphy (LSFG), retinal arteriosclerosis, and the circulation time obtained by fluorescein angiography (FA). *Method*. We evaluated 118 patients and assessed their time of choroidal flush, arm-to-retina time, and early and late phases of retinal circulation time (RT: sec) obtained by FA. The severity of retinal arteriosclerosis was classified according to the Scheie classification. The MBR values throughout the ONH (MBR-A), in the tissue (MBR-T), and in the vessels (MBR-V) were analyzed. *Results*. Patients with retinal vein occlusion (RVO) showed prolonged early and late phases of RT compared to other ocular diseases. Single and multiple regression analyses showed that the MBR-V and Scheie classification were significantly associated with both the choroidal flush and arm-to-retina times. The incidences of RVO and MVR-V were significantly associated with the early phase of RT, and the incidences of RVO, MBR-V, Scheie classification, and gender were revealed to be factors independently contributing to the late phase of RT. *Conclusion*. MBR-V in the ONH and retinal arteriosclerosis are important contributing factors for the circulation time of each stage obtained by FA.

## 1. Introduction

Angiography can be regarded as the classic and standard method for the evaluation of the circulation anywhere in the human body. In the field of ophthalmology, since the development of fluorescein angiography (FA) [1], FA has become the most important method for the evaluation of chorioretinal circulatory disorders. FA gives detailed information about a patient's chorioretinal vascular condition, for example, retinal vein and artery occlusion [2, 3]. FA can also detect capillary abnormalities such as microaneurysms [4].

FA can reflect the hemodynamics of the chorioretinal circulation in detail. The circulation parameters usually used are the time of choroidal flush, the arm-to-retina circulation time, and the retinal circulation time (RT) [5]. It has been suggested that the choroidal flush and the arm-to-retina times reflect the hemodynamics of the posterior ciliary artery and central retinal artery and that the RT represents the passage time from the first influx of fluorescein dye in the retinal arterioles to its arrival in the retinal venules, passing the retinal microcirculation [5]. Thus, RT can serve as a good indicator of retinal microcirculation.

Several studies have shown that the measurement of RT is reproducible and prolonged in ischemic retinal disease and glaucoma [6–9]. It was also suggested that RT is influenced by retinal arteriosclerosis [10]. However, in rare cases, intravenous fluorescein causes severe side effects [11, 12], and thus, it is difficult to perform FA repeatedly for the same patient.

Laser speckle flowgraphy (LSFG) is a noninvasive method for quantifying the ocular blood flow [13, 14]. This method is based on the changes in the speckle pattern of laser light reflected from the fundus of the eye [15, 16]. LSFG depends on the movement of erythrocytes in the retina, the choroid and the optic nerve head (ONH), and the mean blur rate (MBR), which is an indicator of blood flow [17, 18]. In 2008, the LSFG-NAVI™ system (Softcare Co., Fukuoka, Japan) was approved as a medical apparatus by the Pharmaceuticals and Medical Devices Agency in Japan. The MBR is a quantitative index of the blood flow velocity [19–21], and MBR measurements are highly reproducible [22].

Considering these prior findings, we hypothesized that the MBR shown by the LSFG method may reflect each circulation time obtained using FA. The purpose of the present study was thus to determine whether there are significant correlations among the MBR in the ONH and the circulation times obtained using fluorescein angiography. We also evaluated the relationship between each type of circulation time and the retinal arteriosclerosis by Scheie classification [23, 24].

## 2. Materials and Methods

### 2.1. Subjects

The institutional review board of Toho University Omori Medical Center approved this study. All subjects provided informed consent to participate in this research in accord with the Declaration of Helsinki. We studied 118 consecutive subjects who underwent FA at the Department of Ophthalmology, Toho University Omori Medical Center, between September 1, 2015, and March 1, 2016. Patients who had uveitis, definite atrial fibrillation, or a history of intraocular surgery were excluded.

### 2.2. FA Measurements

The circulation times shown by FA were obtained by using an IMAGEnet Digital Imaging System™ (Topcon Co., Tokyo). All patients were evaluated under mydriasis achieved by the administration of eye drops containing 0.5% tropicamide and 0.5% phenylephrine hydrochloride (Mydrin-P, Santen Pharmaceutical, Osaka, Japan). After mydriasis was achieved, 500 mg of fluorescein dye (Fluorescite®, Alcon Japan, Tokyo) was injected rapidly into a catheterized antecubital vein.

We calculated the following phases in the transit of fluorescein in the fundus: (1) the time of the choroidal flush (sec), (2) the arm-to-retina time (sec), which reflects the early arteriolar phase and was recorded for the appearance of the retinal arterial fluorescence, and (3) the early and late phases of the retinal circulation time (RT: sec). The early phase was measured from the point at which the retinal artery was filled with fluorescence to the time point of the relevant vein laminar flow outset. The late phase was measured from the point at which the retinal artery was filled with fluorescence to the time at which the relevant vein was completely filled. Photographs of the fundus image were obtained at 1 sec intervals until the late phase of RT.

We divided the subjects' diseases into diabetic retinopathy (DR), age-related macular degeneration (AMD), retinal vein occlusion (RVO), and “others.”

### 2.3. The Scheie Classification

The extent and severity of arteriosclerotic change in the retinal arteries were classified according to the following definition [23, 24]. Group 1 was defined as a broadening of the light reflex from the artery, with minimal or no arteriovenous compression. Group 2 was defined as the changes similar to those in stage 1, but more prominent. In group 3, the arteries have a “copper wire” appearance, the arteriovenous compression is much greater, and serious atherosclerotic changes of the retinal arteries are present. In group 4, the arteries have a “silver wire” appearance, and the arteriovenous crossing changes are the most severe.

The upper temporal quadrants and the first three arterial branchings were analyzed in all patients. The eye fundus examinations were carried out by fundus photography by the same ophthalmologist (H. O.) who had no medical information about the patients. The study eye was chosen as the side opposite the eye used for the FA.

### 2.4. LSFG Measurements in the ONH

**Figure 1:**
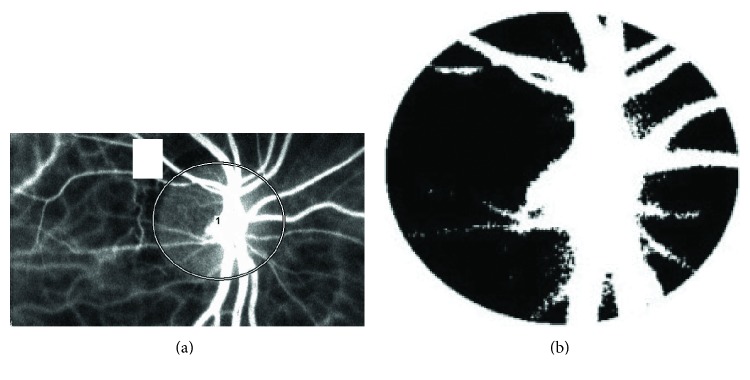
(a) The margin in the ONH obtained by hand using a round band. (b) The components of vessels and tissues were separated out using the automated definitive threshold.

The determination of LSFG from ONH images has been described in detail [18, 25]. In the present study, we used the MBR as an indicator of blood flow in the LSFG measurements. We calculated the three parameters of the MBR in the ONH described below by using the LSFG Analyzer software (v.3.0.47, Softcare). After we had identified the margin of the ONH by hand using a round band (Figure 1(a)), the software separated out the vessels using the automated definitive threshold (Figure 1(b)) and then analyzed the means of the MBRs in the vessels of the ONH (MBR-V), in the ONH tissue (MBR-T), and throughout the ONH (MBR-A) [21, 22, 26]. The eye studied was the same side as that used for the FA evaluation. The LSFG measurements were made immediately prior to the FA evaluation and were obtained after the subject rested for 10 min in a quiet room maintained at 24°C, with mydriasis. We measured the ONH three times and used the average for the statistical analyses.

### 2.5. Measurements of Other Parameters

The following parameters were recorded: history of diabetes mellitus, hypertension, and the use of anticoagulant agents. Systolic blood pressure (SBP, mmHg), diastolic blood pressure (DBP, mmHg), heart rate (beats per min, bpm), mean arterial blood pressure (MABP, mmHg), intraocular pressure (IOP, mmHg) measured by applanation tonometry, ocular perfusion pressure (OPP, mmHg), and spherical refraction (Ref: diopters, D) were assessed with the TONOREF 2™ system (Nidek Co., Aichi, Japan).

Diabetes mellitus was diagnosed in the subjects who were already undergoing treatment for diabetes mellitus, and we diagnosed hypertension if the subject was receiving antihypertensive therapy.

The MABP was determined by the following formula: DBP + (SBP − DBP)/3.

The OPP was defined as (2/3MABP) − IOP.

All parameters were evaluated at the same time as the LSFG measurements.

### 2.6. Statistical Analysis

The data are presented as the means ± standard deviation (SD) for the continuous variables. We performed one-way analysis of variance (ANOVA) to determine whether there is a difference in each type of circulation time revealed by FA in the different diseases. We used single regression analysis to determine the relationship between each type of circulation time revealed by FA, the Scheie classification, and the values of MBR-V, MBR-T, and MBR-A in the ONH and other parameters. We used a multiple regression analysis to determine the independent factors for each type of circulation time revealed by FA. *p* values < 0.05 were considered significant. The StatView v 5.0 program (SAS Institute, Cary, NC) was used for the statistical analyses.

## 3. Results

Table 1 summarizes the characteristics of the 118 (84 men, 34 women) patients. The mean ± SD age was 60.4 ± 15.1 yrs. Thirty-six patients had DR, 19 patients had AMD, 26 patients had RVO, and 37 patients had other diseases. Table 2 summarizes the circulation time results (i.e., time of choroidal flush, arm-to-retina time, and early and late phases of RT), the retinal arteriosclerosis (Scheie classification), and the MBRs in the ONH obtained by LSFG.

The Scheie classifications were as follows: normal: 31 (26.3%) patients; group 1: 61 (51.7%) patients; group 2: 22 (18.6%) patients; group 3: four (3.4%) patients; and group 4: 0 (0%) patients. Table 3 shows the FA-measured circulation times for each disease. The early and late phases of RT were significantly longer in the RVO patients than in the patients with DR, AMD, and other diseases (by one-way ANOVA followed by Fisher's PLSD post hoc test, *p* = 0.005), respectively.

Table 4 shows the correlation between each circulation time in FA and the retinal arteriosclerosis by Scheie classification. Time of choroidal flush (*r* = 0.26, *p* = 0.005), arm-to-retina time (*r* = 0.28, *p* = 0.003), and the late phase of RT (*r* = 0.32, *p* = 0.0004) were significantly positively correlated with the progression of Scheie classification.

Table 5 shows the results of the single regression analysis of the relationships among each circulation time in FA and the three sections of MBR in the ONH. The choroidal flush and arm-to-retina times were significantly negatively correlated with MBR-V (*r* = −0.37, *p* < 0.0001; *r* = −0.37, *p* < 0.0001) and MBR-A (*r* = −0.27, *p* = 0.003; *r* = −0.25, *p* = 0.006), respectively. The early and late phases of RT were significantly negatively correlated with MBR-V (*r* = −0.42, *p* < 0.0001; *r* = −0.35, *p* = 0.0001), MBR-T (*r* = −0.34, *p* = 0.0002; *r* = −0.24, *p* = 0.01), and MBR-A (*r* = −0.39, *p* < 0.0001; *r* = −0.25, *p* = 0.008), respectively.

The results of the single regression analysis between each type of circulation time in FA and the subjects' clinical characteristics are shown in Table 6. Gender (men = 1, women = 0) was correlated significantly with the early phase (*r* = 0.19, *p* = 0.04) and late phase (*r* = 0.21, *p* = 0.02) of RT and tended to be correlated with the choroidal flush (*r* = 0.17, *p* = 0.07) and arm-to-retina times (*r* = 0.15, *p* = 0.10) but the correlation did not reach significance. Diabetes mellitus tended to be correlated with the choroidal flush (*r* = 0.15, *p* = 0.11) and the arm-to-retina times (*r* = 0.18, *p* = 0.06). Hypertension was significantly correlated with the choroidal flush (*r* = 0.20, *p* = 0.03) and arm-to-retina times (*r* = 0.23, *p* = 0.01) and tended to be correlated with the early phase (*r* = 0.16, *p* = 0.09) and the late phase (*r* = 0.17, *p* = 0.07) of the RT.

Table 7 shows the results of the multiple regression analysis for factors independently contributing to the time of the choroidal flush and the arm-to-retina time. The MBR-V and Scheie classification were identified as factors contributing independently to both the choroidal flush and arm-to-retina times (time of choroidal flush: MBR-V, standard regression = −0.32, *p* = 0.0003; Scheie classification, standard regression = 0.19, *p* = 0.04; arm-to-retina time: MBR-V, standard regression = −0.31, *p* = 0.0005; Scheie classification, standard regression = 0.20, *p* = 0.03).

We also conducted a multiple regression analysis to identify independent contributors to the early and late phases of RT (Table 8). The MBR-V (standard regression = −0.36, *p* < 0.0001) and RVO (standard regression = 0.31, *p* = 0.0002) were identified as factors contributing independently to the early phase of RT. RVO (standard regression = 0.32, *p* = 0.0001), MBR-V (standard regression = −0.26, *p* = 0.001), Scheie classification (standard regression = 0.25, *p* = 0.003), and gender (men = 1, women = 0, standard regression = 0.19, *p* = 0.02) were all identified as factors contributing independently to the late phase of RT.

## 4. Discussion

Fluorescein angiography is useful for understanding the hemodynamics of the chorioretinal circulation in detail. As noted in the Introduction, intravenous fluorescein can cause severe side effects [11, 12], and it may thus be unwise to conduct FA repeatedly for the same patient. Other noninvasive methods for predicting the ocular circulation time are desirable. LSFG is a noninvasive quantitative method for determining the ocular blood flow [13, 14], and the MBR, an LSFG parameter, is a quantitative index of the blood flow velocity [19–21].

A few research groups reported that the MBR-V in the ONH reflected the early phase of RT [27, 28], but these studies examined only central RVO cases. It thus remains unclear whether the mean blur rate in the optic nerve head as measured by LSFG reflects the circulation time obtained by FA in different ocular diseases. The relationships between the MBR and circulation times other than those in the early phase of RT also remain to be elucidated.

Our goal in the present study was thus to determine whether there are significant correlations among the MBR in the ONH, the retinal arteriosclerosis by Scheie classification, and the circulation time obtained by FA, in a variety of ocular diseases of numerous patients. Our results demonstrated that the patients with RVO had prolonged early and late phases of RT compared to the patients with other ocular diseases. Previous studies revealed that the measurement of RT is prolonged in patients with RVO, which is one of the microcirculation disorders [7, 10, 29]. Our present findings reconfirmed that the congested retinal venules due to RVO affected the retinal microcirculation shown by RT.

Our single regression analysis showed that the Scheie classification was significantly positively correlated with the time of choroidal flush, the arm-to-retina time, and the late phase of RT. In other words, the time of choroidal flush, arm-to-retina time, and late phase of RT may be prolonged in parallel with the exacerbation of retinal arteriosclerosis.

The results of the single regression analysis also revealed that the choroidal flush and arm-to-retina times were significantly negatively correlated with the MBR-V and MBR-A and that each phase of the RT was significantly negatively correlated with all sections of the MBR. Among them, the MBR-V was most strongly correlated with all of the circulation times shown by FA. This suggests that the circulation times shown by FA may be prolonged in parallel with the decrease in the MBR-V.

Our multiple regression analysis showed that the MBR-V and retinal arteriosclerosis by Scheie classification were significantly associated with the choroidal flush and arm-to-retina times. The MBR-V, in particular, contributed most strongly to the choroidal flush and arm-to-retina times.

Our results also confirmed that the MBR-V and retinal arteriosclerosis may be important components of the hemodynamics of the posterior ciliary artery and the central retinal artery, regardless of the type of ocular diseases.

The multiple regression analysis showed that RVO and MVR-V were significantly associated with the early phase of RT. Moreover, RVO, the MBR-V, the Scheie classification, and gender were also revealed to be factors independently contributing to the late phase of RT, suggesting that RVO and MBR-V may more strongly influence the RT, which is an indicator of retinal microcirculation.

On the other hand, the Scheie classification and gender were also significantly correlated with the late phase of RT. We speculated that the difference in length and weight due to gender differences may affect the late phase of RT. In addition to the MBR-V, retinal arteriosclerosis may be an important factor contributing to the microcirculation shown by RT. Ophthalmologists should pay attention to the importance of Scheie classification not only in light of systemic arteriosclerosis but also concerning ocular microcirculation.

We reported that the secretion levels of vasoactive mediators were significantly correlated with the MBR-V [21]. The MBR-V, representing the vascular component, is considered to be suitable for evaluating intravessel hemodynamics, such as circulation times. Based on our results, we speculate that the combined analysis of the status of the MBR-V and retinal arteriosclerosis may someday be used to predict to some extent the circulation times obtained by FA, which is an invasive method.

Our study has some major limitations. First, there are some problems with the evaluation method of FA. The dye was injected rapidly into a catheterized antecubital vein in all subjects, but there are individual differences in the distance from the arm to the eye, and we did not examine these differences. A reanalysis after adjusting the height, weight, and distance from the arm to the eye is necessary. Second, an administration of eye drops containing phenylephrine was used for mydriasis for the FA. The phenylephrine might have affected the outcome of the MBR-V measurements.

In addition, we did not evaluate the ocular circulation divided into the individual type (branch or central RVO) or severity of diseases. Further careful validation evaluations are necessary, in accord with the type and severity of various ocular diseases. However, in the present study, a power analysis (linear multiple regression) by G∗Power software (v.3.1.3; developed by Franz Faul, Kiel University, Kiel, Germany) showed that a total sample size of 102 is needed for an effect size *f*^2^ of 0.15, an error probability (*a*) of 0.05, and a power (1 − *b*) of 0.85. Therefore, the total sample size of 118 in the present study is a sufficient sample size.

In conclusion, MBR-V representing the vascular component in the optic nerve head as shown by LSFG is an important contributing factor for the circulation time of each stage obtained by fluorescein angiography. Retinal arteriosclerosis may also be an important factor, in the same way.

## Figures and Tables

**Table 1:** Clinical characteristics of the 118 study patients.

Men : women	84 : 34
Age (yrs)	60.4 ± 15.1
Diabetes mellitus (%)	43 (36.4)
Hypertension (%)	50 (42.4)
Anticoagulant agents (%)	10 (8.5)
SBP (mmHg)	142 ± 20
DBP (mmHg)	82 ± 14
Heart rate (beat per min)	78 ± 14

Target eye of	

Ref (D)	−1.2 ± 2.8
IOP (mmHg)	14.1 ± 3.1
OPP (mmHg)	53.9 ± 9.7

Diseases	

DR (%)	36 (30.5)
AMD (%)	19 (16.1)
RVO (%)	26 (22.0)
Others (%)	37 (31.4)

The data are mean ± SD or number of patients (%). SBP: systolic blood pressure, DBP: diastolic blood pressure, Ref: spherical refraction, IOP: intraocular pressure, OPP: ocular perfusion pressure, DR: diabetic retinopathy, AMD: age-related macular degeneration, and RVO: retinal vein occlusion.

**Table 2:** Circulation times obtained by FA, arteriosclerosis, and ONH circulation shown by laser speckle flowgraphy.

Fluorescein angiography (FA)	

Time of choroidal flush (sec)	16.0 ± 4.4
Arm-to-retina time (sec)	18.5 ± 4.5
RT: early phase (sec)	3.4 ± 1.7
RT: late phase (sec)	11.8 ± 4.0

Scheie classification	

Normal (%)	31 (26.3)
Group 1 (%)	61 (51.7)
Group 2 (%)	22 (18.6)
Group 3 (%)	4 (3.4)
Group 4 (%)	0 (0)

LSFG	

MBR-V	38.3 ± 7.5
MBR-T	10.9 ± 2.4
MBR-A	20.3 ± 4.3

The data are mean ± SD or number of patients (%) (*n* = 118).

RT: retinal circulation time, LSFG: laser speckle flowgraphy, and MBR: mean blur rate.

**Table 3:** Circulation times in each disease as shown by FA.

	DR	AMD	RVO	Others	*p* value
Time of choroidal flush	16.1 ± 3.7	15.9 ± 4.9	15.4 ± 4.2	16.7 ± 4.7	0.70
Arm-to-retina time	18.7 ± 3.6	18.1 ± 5.3	17.9 ± 4.3	19.0 ± 5.1	0.78
RT: early phase	3.5 ± 1.8	3.2 ± 1.5	4.5 ± 2.0^†,‡^	2.9 ± 1.3	0.005
RT: late phase	11.4 ± 3.9	10.8 ± 3.9	14.4 ± 4.2^∗^	11.0 ± 3.3	0.005

The data are mean ± SD. One-way ANOVA followed by Fisher's PLSD post hoc test. RT: retinal circulation time, DR: diabetic retinopathy, AMD: age-related macular degeneration, and RVO: retinal vein occlusion.

^†^Versus DR, *p* < 0.05; ^‡^versus AMD and others, *p* < 0.01.

^∗^Versus DR, AMD, and others, *p* < 0.01.

**Table 4:** Correlation between circulation times in FA and ocular arteriosclerosis shown by Scheie classification.

Explanatory variable	*r* value	*p* value
Time of choroidal flush	0.26	0.005
Arm-to-retina time	0.28	0.003
RT: early phase	0.13	0.18
RT: late phase	0.32	0.0004

Objective variables = Scheie classification (normal to group 3).

Single regression analysis. RT: retinal circulation time.

**Table 5:** Correlation between circulation times in FA and MBR values shown by LSFG.

Explanatory variable	MBR-V		MBR-T		MBR-A	
	*r*	*p*	*r*	*p*	*r*	*p*
Time of choroidal flush	−0.37	<0.0001	−0.11	0.24	−0.27	0.003
Arm-to-retina time	−0.36	<0.0001	−0.08	0.40	−0.25	0.006
RT: early phase	−0.42	<0.0001	−0.34	0.0002	−0.39	<0.0001
RT: late phase	−0.35	0.0001	−0.24	0.01	−0.25	0.008

Single regression analysis. MBR: mean blur rate and RT: retinal circulation time (*n* = 118).

**Table 6:** Results of single regression analysis between the circulation times in FA and clinical characteristics.

Explanatory variable	Time of choroidal flush		Arm-to-retina time		RT: early phase		RT: late phase	
	*r*	*p*	*r*	*p*	*r*	*p*	*r*	*p*
Men = 1, women = 0	0.17	0.07	0.15	0.10	0.19	0.04	0.21	0.02
Age	0.10	0.29	0.11	0.24	0.02	0.84	−0.01	0.88
Diabetes mellitus (+ = 1, − = 0)	0.15	0.11	0.18	0.06	0.02	0.85	−0.07	0.46
Hypertension (+ = 1, − = 0)	0.20	0.03	0.23	0.01	0.16	0.09	0.17	0.07
Anticoagulant agents (+ = 1, − = 0)	−0.02	0.84	0.02	0.82	0.12	0.21	−0.08	0.40
SBP	<−0.01	0.99	0.02	0.86	0.08	0.39	−0.11	0.22
DBP	−0.03	0.72	−0.03	0.78	0.15	0.11	−0.02	0.83
Heart rate	−0.10	0.30	−0.07	0.45	0.04	0.64	<−0.01	0.99
Ref	0.04	0.70	0.04	0.68	−0.03	0.77	−0.06	0.52
IOP	0.03	0.76	−0.01	0.91	−0.01	0.95	0.04	0.65
OPP	−0.03	0.74	−0.01	0.95	0.13	0.15	−0.08	0.39

RT: retinal circulation time; Ref: spherical refraction, IOP: intraocular pressure, and OPP: ocular perfusion pressure (*n* = 118).

**Table 7:** Results of multiple regression analysis for factors independently contributing to the time of the choroidal flush and the arm-to-retina time (*n* = 118).

Explanatory variable	Time of choroidal flush		Arm-to-retina time	
	Standard regression	*p*	Standard regression	*p*
MBR-V	−0.32	0.0003	−0.31	0.0005
Scheie classification (normal to group 3)	0.19	0.04	0.20	0.03
Men = 1, women = 0	0.08	0.34	0.06	0.48
Diabetes mellitus (+ = 1, − = 0)	0.09	0.31	0.11	0.19
Hypertension (+ = 1, − = 0)	0.06	0.51	0.09	0.33

Correlation coefficients: *r* = 0.46, *p* < 0.0001; *r* = 0.47, *p* < 0.0001.

**Table 8:** Results of multiple regression analysis for factors independently contributing to the retinal circulation time (*n* = 118).

Explanatory variable	RT (early phase)		RT (late phase)	
	Standard regression	*p*	Standard regression	*p*
RVO (+ = 1, − = 0)	0.31	0.0002	0.32	0.0001
MBR-V	−0.36	<0.0001	−0.26	0.001
Scheie classification (normal to group 3)			0.25	0.003
Men = 1, women = 0	0.14	0.08	0.19	0.02
Hypertension (+ = 1, − = 0)	0.08	0.31	0.02	0.81

Correlation coefficients: *r* = 0.54, *p* < 0.0001; *r* = 0.57, *p* < 0.0001.

RT: retinal circulation time, RVO: retinal vein occlusion, and MBR: mean blur rate.
